# Nursing-centered development of an AI-based decision support system in pressure ulcer and incontinence-associated dermatitis management - a mixed methods study

**DOI:** 10.1186/s12912-025-03448-4

**Published:** 2025-07-01

**Authors:** Khalid Majjouti, Vanessa Priester, Michaela Tapp-Herrenbrueck, Alexander Brehmer, Hannah Pinnekamp, Michael Aleithe, Uli Fischer, Jens Kleesiek, Bernadette Hosters

**Affiliations:** 1 Department of Nursing Development and Nursing Research, University Hospital Essen, Essen, Germany; 2 Institute for Artificial Intelligence in Medicine (IKIM), University Hospital Essen, Essen, Germany; 3 Department of Clinical Nursing Research and Quality Management, Nursing Department, Hospital of the Ludwig Maximilian University (LMU) Munich, Munich, Germany; 4 sciendis GmbH, Leipzig, Germany

**Keywords:** Pressure ulcer prevention, Pressure injury, Incontinence-associated dermatitis, Artificial intelligence, Decision-making support system, Wound management, Evidence-based care

## Abstract

**Background:**

Differentiating between stage 1 or 2 pressure ulcer/pressure injury (PU/PI) and incontinence-associated dermatitis (IAD) poses a significant challenge for healthcare professionals, due to their visual similarity. Incorrect assessments may trigger inappropriate interventions, potentially resulting in delayed treatment. KIADEKU is a multi-center research project aimed at supporting the assessment and documentation of PU/PI and IAD, as well as the implementation of evidence-based care through an AI-based application in nursing care. This paper investigates how to integrate evidence from nursing science and clinical practice into the development of the proposed AI system.

**Methods:**

We conducted a literature review of nursing criteria for wound assessment. Nursing experts iteratively evaluated the findings, leading to the definition of a Minimum Data Set (MDS) that the research team used to annotate wound images for AI training. We collected a data set of wound images from the medical records of two university hospitals. To ensure high data quality, we implemented a validation process involving up to four independent expert assessments of each wound image. We calculated Krippendorff’s alpha to assess the internal consistency of the annotation process for reliability analysis. This study adhered to the TRIPOD-AI guidelines.

**Results:**

The differentiation between PU/PI and IAD primarily relies on clinical observation and visual inspection, with key factors including aetiology, anatomical location, and wound morphology. The validated MDS encompasses 18 wound-related and four aetiological categories, including visual and contextual patient data. The AI system consequently integrates wound images with categorical patient information. The reliability analysis of 1,521 annotated wound images indicates substantial agreement for wound type classification (α = 0.64, 95% CI 0.62–0.68) and fair to moderate agreement for PU/PI (α = 0.57, 95% CI 0.55–0.63) and IAD categorization (α = 0.27, 95% CI 0.20–0.36).

**Conclusions:**

The integration of evidence from nursing science and practice into the AI development process using a mixed-methods approach, established a robust, evidence-based foundation. This approach yielded an innovative implementation of routine care data for AI training, advancing the field of AI-driven wound care solutions.

**Trial registration:**

Registered with the German Clinical Trials Register (DRKS) on 2023–09-05. DRKS-ID: DRKS00029961.

## Background

Differentiating between pressure ulcers (PU) and incontinence-associated dermatitis (IAD) poses a significant challenge even for experts in the field of wound care [1–4]. PU is defined as ‘localised damage to the skin and/or underlying tissue, typically over bony prominences, as a result of pressure or pressure combined with shear forces’ [5]. In 2016, the National Pressure Ulcer Advisory Panel introduced updated guidelines, recommending the term ‘pressure injury’ (PI) to better encompass all forms of tissue damage, including stages before visible skin breakdown [6]. While this shift reflects a broader understanding of the condition, it appears that both terms have limitations [7]. Currently, in Europe, the term ‘pressure ulcer’ remains widely used [8]. In this article, we will use the terms pressure ulcer and pressure injury interchangeably. IAD is an irritant contact dermatitis in adults caused by extended exposure to urine and/or faeces [9]. Similarities in the clinical presentation can confuse accurate diagnosis. Both diseases initially produce clearly defined erythema. They can also develop at the same time or contribute to each other. As a result, PU/PI and IAD are often mistaken in practice [9–11]. Globally, the mean prevalence of PU/PI was 14.84% and ranged from 2.20% to 40.0% across different populations [12]. International studies estimate the prevalence of IAD in long-term and acute care settings to be up to 45.7% [13]. PU/PI and IAD can cause extremely painful conditions, leading to physical and mental suffering including depression and loss of independence [14, 15]. Furthermore, they are associated with a risk of serious complications, e.g. secondary infections [2, 16]. The prevention and treatment of PU/PI and IAD also represent a significant economic burden for healthcare systems [17–19]. There are no agreed international clinical diagnostic criteria to distinguish IAD from other skin conditions, including PU/PI [9]. Currently, there is no Point-of-Care technology available to assist the assessment and diagnosis of IAD at the bedside. Accurate diagnosis is essential, as their primary causes and treatments differ. Treating the underlying cause is a key factor to successful therapy [13]. The therapeutic approach to IAD emphasizes skin protection [4], whereas PU/PI management prioritizes pressure reduction [5]. An incorrect assessment can lead to delayed wound healing or even deterioration caused by inappropriate interventions.

Artificial Intelligence (AI) is considered a promising approach to improve the quality of wound management, including patient safety, clinician time and cost-effectiveness [20–22]. In PU/PI management, AI technology can help enhance risk assessment, prevention, diagnosis and classification [23]. Seibert et al. report on a range of AI approaches in healthcare, including risk assessment and PU/PI management. However, their findings reveal a lack of evidence on the application of AI systems in real-world scenarios, as well as research with a nursing care-specific perspective [24].

KIADEKU^1^ is a research and development project aimed at supporting the assessment and documentation of PU/PI and IAD, as well as the implementation of evidence-based care through an image-based AI application in nursing care [25]. The developed AI system supports the differentiation between PU/PI and IAD, and predicts classification and other wound characteristics. It provides objective assessment and categorization to improve accuracy of assessment and documentation. To support decision-making, we developed a complementary decision matrix to link predictions to evidence-based care workflows. The University Hospital Essen, the Hospital of the Ludwig Maximilian University Munich and the Institute for Artificial Intelligence in Medicine at the University Medicine Essen cooperated on the development of the AI system, while sciendis Ltd., a software company in the medical sector, was responsible for building the application. In the KIADEKU project, we iteratively evaluate the development process through a Delphi study at five survey points, each with a different focus. This interdisciplinary approach ensures that the project benefits from advanced practical expertise in nursing and medicine, AI research and medical technology. This paper investigates how evidence from nursing science and clinical practice can be integrated into the development of the proposed AI system.

## Methods

We employed a mixed methods design, combining quantitative and qualitative data collection and analysis strategies. Our approach includes a modified Delphi Survey for the development of a Minimum Data Set (MDS) for the classification and categorization of PU/PI and IAD. We performed a reliability analysis to assess the internal consistency of the MDS.

## Aim of the study

This paper presents a sub-study of the KIADEKU project that aims to facilitate the development of the proposed AI system by systematically synthesizing and integrating evidence from nursing science, clinical practice, and expert knowledge in the validation of wound images for AI training. The study focuses on defining and validating the MDS and applying it to annotate wound images, essential for preparing a robust, evidence-based foundation.

## Design

### Literature and documentation analysis

Initially, we compared and analyzed the electronic documentation systems employed by the participating university hospitals. Additionally, we conducted a systematic literature review to identify nursing criteria for the assessment and differentiation of PU/PI and IAD. This review focused on nursing guidelines and expert standards since 2019 in German or English, which explicitly addressed PU/PI or IAD. Following databases were searched for guidelines:

- Centre for Quality in Nursing (ZQP) database of nursing guidelines and standards,
- Programme for National Nursing Guidelines (NVL),
- Guidelines International Network (–), and
- National Guideline Clearinghouse.

Furthermore, we included the databases Medline (via PubMed), CINAHL and Cochrane Library. The search strategy was guided by the specific research principle outlined in the RefHunter protocol [26].

### Validation and annotation process

The findings were discussed in an expert panel comprising nursing scientists, dermatologists, and medical computer scientists. The panel evaluated the identified features for relevance and feasibility, consolidating them into a MDS. The MDS included wound characteristics as well as health care information. In the next stage, we assessed the validity and practicability of the results as part of the first survey point in the Delphi study with nursing practitioners. The full analysis of the Delphi study will be published separately.

We utilized the revised MDS (Table 2 and 3) to annotate wound images with Label Studio software. The images derived from the medical records of the two university hospitals in the project, captured in routine care from 2009 to 2023. We included only wound images of the pelvic and gluteal area from patients aged 18 years and older, identified through the existing reporting systems at the respective sites, after preselecting for photo quality, resulting in a final inclusion of 1,521 wound images. Thus, the data set for AI training consisted of wound images collected from real-world clinical scenarios. Data processing procedures were conducted in accordance with applicable laws and guidelines. The annotations were completed in four successive batches. Both university hospitals cover the whole range of modern healthcare and medicine in Germany.

The annotation process involved four wound experts and nurse scientists with extensive practical experience. As defined in the MDS we annotated wound characteristics and health care information, for example mobility and continence. For wound categorization we applied the International Classification of Diseases, 10th Revision (ICD-10-GM) for PU [27], which is the prevailing classification system in German hospitals at present, and Ghent Global IAD Categorization Tool (GLOBIAD) for IAD [9], both internationally recognized standards. Three of the experts independently annotated each wound image without consulting the original medical record, except for non-photographic features (Table 3). We calculated Krippendorff’s alpha [28] with associated 95% confidence intervals (CI) to assess the internal consistency of the annotation process for reliability analysis using the statistical software IBM SPSS Statistics 29.0. We followed the most common interpretation guideline (0.00–0.20: slight, 0.21–0.40: fair, 0.41–0.60: moderate, 0.61–0.80: substantial, 0.81–1.00: almost perfect agreement) [29, 30]. This was concluded by a consensus discussion to resolve discrepancies between the experts’ assessments, with a fourth expert opinion being obtained if necessary.

The results provided the evidence for the development and evaluation of a multimodal deep learning framework. Several state-of-the-art models were tested, to optimize for performance and computational efficiency in the clinical setting. To further improve performance, varying data processing strategies were systematically evaluated, including different combinations of various training, augmentation, data pre- and post-processing methods. The details of development and technical analysis have recently been accepted for publication and are currently undergoing editorial review [31].

This study adhered to the TRIPOD-AI guidelines [32].

## Results

### Evidence synthesis

The comparative analysis of the electronic documentation systems employed by the participating university hospitals revealed notable differences between the sites. In particular, the documentation of PU/PI was found to be based on disparate classification systems, with one site utilizing the ICD-10-GM [27] and the other employing the European Pressure Ulcer Advisory Panel (EPUAP) [5] framework. Furthermore, neither hospital had implemented a standardized assessment instrument or a structured data format for the documentation of IAD.

In the literature review, we were unable to identify any new recommendations for the categorization of PU/PI. Current classification systems are similar, with minor variations in terminology and definitions. Clinicians are advised to use the system adopted by their healthcare organization to ensure consistent and comparable reporting [5, 7, 27, 33–35].

Our review revealed a best practice recommendation for IAD management developed by the umbrella organization of German-speaking wound treatment societies (Wund-D.A.CH.) [13]. Their recommendations are based in the GLOBIAD Categorization Tool [9]. The GLOBIAD tool provides a systematic framework for assessing IAD. IAD is characterized by skin redness (Category 1), and skin loss (Category 2). Each category is further subdivided into A and B based on the absence or presence of clinical infection signs, resulting in a total of four distinct categories.

The differentiation between PU/PI and IAD is based on clinical observation and visual inspection. The primary factors to consider for differentiation include aetiology, anatomical location, and wound morphology (Table 1) [3, 36].

**Table 1:** Differentiation between PU/PI and IAD

Parameter	PU/PI	IAD
Anamnesis	Exposure to pressure/shear forces, immobility, reduced sensitivity	Urinary and/or faecal incontinence
Symptom	Pain	Pain, burning, itching, tingling
Anatomical Location	Usually over a bony prominence.	perineum, perigenital areas, buttocks, gluteal sulcus, medial and posterior thighs, lower back, can extend over a bony prominence
Shape/Edges	distinct edges	Affected area is diffuse with poorly defined edges/may be patchy.
Presentation/Depth	Presentation varies from intact skin with non-blanchable erythema to complete skin loss. Wound base may contain necrotic tissue.	Intact skin with erythema (blanchable or non-blanchable), with/without superficial, partial thickness skin loss.
Other	Secondary soft tissue infection may occur.	Secondary superficial skin infection (e.g. candidiasis) may occur. Periwound area usually macerated.

Based on the analysis of the documentation systems and the literature evidence, the expert panel defined a MDS, which we validated in the Delphi survey. The validated MDS encompasses 18 wound-related features and 4 aetiological categories. The MDS and attributes for each category are detailed in Tables 2 and 3.

**Table 2:** Minimum data set excluding non-photographic features

Category	Attributes
Anatomical Location	glutes anal fold genital area sacrum trochanter groin unilateral/both sides anterior thigh unilateral/both sides posterior thigh unilateral/both sides
Wound type	PU/PI IAD both not assessable/none
Categorization	PU/PI according to ICD-10 IAD according to GLOBIAD
Wound bed	fibrin granulation necrosis muscle/fascia tendon bone fat tissue dermis erythema not assessable
Wound bed characteristics	moist dry glossy not assessable
Wound edge	smooth bulging undermined hyperkeratotic vital rolled (epibole) reddish macerated livid not assessable
Wound morphology	diffuse round/oval distinct edges none
Periwound area	dry moist cracked oedematous eczematous macerated blistering intact atrophic none
Periwound colour	livid flushed brown none

**Table 3:** Minimum data set non-photographic features

Category	Attributes
Wound Size	length width depth surface area
Wound Pockets	yes localisation no
Pain	yes no not assessable
Itching	
Odor	
Exudate level	none low moderate plenty not assessable
Exudate quality	purulent not purulent not assessable
Exudate colour	pink/red green turbid serous bloody yellow brown not assessable
Signs of infection	redness/Rubor overheated/Calor swelling/Tumour pain/Dolor functional impairment/Functio Laesa none
Mobility	absent mostly absent mostly complete complete not assessable
Urinary continence	absent mostly absent mostly complete complete not assessable
Feacal continence	
Sensory ability	

## Validation and annotation

We systematically annotated a data set of 1,521 wound images in four batches based on the MDS. From this data set, 1,513 images were validated through consensus among the annotators, while in eight cases, consensus could not be reached. All non-photographic features (Table 3) were extracted from the original medical record in the last step of the annotation process.

The images varied in camera positioning in respect of angle and distance. The wounds depicted exhibited heterogeneous wound characteristics, with various stages of healing, infection, and different tissue. Some images may feature information cards or the clinician’s hand. The majority of images represented patients with light skin tones. The distribution of the data set across wound types and categories after consensus validation is shown in Table 4. A category was not assigned if consensus was reached only for the wound type and not for the category. Wound images labeled as ‘both wound types’ or ‘not assesseable/none’ were excluded from the AI training data set.

**Table 4:** Summary of validated data set

Label	Category	n
**PU/PI** (n = 763)	1	25
	2	187
	3	503
	4	27
	none	21
**IAD** (n = 339)	1A	45
	1B	57
	2A	105
	2B	120
	none	12
**Excluded** (n = 411)	both wound types	143
	not assessable/none	268
	**Total**	**1513**

We assessed the interrater reliability for wound type classification, PU/PI and IAD categorization. The corresponding reliability coefficients among annotators across all batches were α = 0.64 (95% CI 0.62–0.68) for wound type classification, α = 0.57 (95% CI 0.55–0.63) for PU/PI categorization, and α = 0.27 (95% CI 0.20–0.36) for IAD categorization. Images depicting PU/PI and IAD simultaneously were labelled as ‘both wound types’. When excluding ‘both wound types’ responses, agreement for wound type classification improved to α = 0.83 (95% CI = 0.81–0.87). The details of the reliability analysis for all batches and overall are listed in Table 5.

**Table 5:** Interrater Reliability (Krippendorff’s Alpha (α), CI 95%). Batch 1 to 4 and overall

Batch	Wound type classification		PU/PI categorization	IAD categorization
		‘both wound types’ excluded		
**#1** (n = 314)	α = 0.70 (0.52–0.77) (n = 233)	α = 0.89 (0.73–0.94) (n = 220)	α = 0.55 (0.45–0.65) (n = 174)	α = − 0.01 (−0.30–0.19) (n = 21)
**#2** (n = 341)	α = 0.61 (0.56–0.68) (n = 310)	α = 0.74 (0.68–0.84) (n = 269)	α = 0.61 (0.53–0.68) (n = 218)	α = 0.27 (0.15–0.55) (n = 38)
**#3** (n = 600)	α = 0.65 (0.62–0.71) (n = 496)	α = 0.86 (0.85–0.92) (n = 434)	α = 0.53 (0.47–0.60) (n = 298)	α = 0.34 (0.20–0.42) (n = 117)
**#4** (n = 265)	α = 0.36 (0.20–0.55) (n = 74)	α = 0.50 (0.27–0.71) (n = 54)	α = 0.62 (0.31–1.00) (n = 17)	α = 0.19 (−0.09–0.48) (n = 25)
**Overall** (n = 1521)	α = 0.64 (0.62–0.68) (n = 1113)	α = 0.83 (0.81–0.87) (n = 977)	α = 0.57 (0.55–0.63) (n = 707)	α = 0.27 (0.20–0.36) (n = 201)

In addition, we assessed the intrarater reliability for two of the raters on a subset of the data. This subset consisted solely of PU/PI or IAD wound images validated through the consensus discussion. The analysis revealed substantial to almost perfect agreement for wound type classification, with alpha coefficients of 0.78 and 0.81 for the two raters, respectively. For PU/PI categorization, the agreement was moderate to substantial, with alpha coefficients of 0.54 and 0.62. IAD categorization showed the lowest agreement, with 0.47 and 0.26. Detailed results are presented in Table 6.

**Table 6:** Intrarater Reliability (Krippendorff’s Alpha (α), CI 95%)

	Wound type classification	PU/PI categorization	IAD categorization
**Rater 1** (n = 98)	α = 0.78 (0.64–0.90) (n = 98)	α = 0.54 (0.33–0.73) (n = 62)	α = 0.47 (0.18–0.76) (n = 25)
**Rater 2** (n = 130)	α = 0.81 (0.70–0.90) (n = 130)	α = 0.62 (0.43–0.78) (n = 67)	α = 0.26 (0.09–0.47) (n = 46)

## Discussion

While wound data sets share comparable wound criteria, the attributes within each category often differ. The variability in existing wound data sets and their attributes underlines the complexity of standardizing wound documentation [37, 38]. For example, there is no evidence whether reporting the amount of exudate in three (none, low, plenty) or four levels (none, low, moderate, plenty) makes a difference in terms of validity and consistency [38, 39]. Given this uncertainty, it is important to advise clinicians to adhere to the reporting system in place in their healthcare organization to maintain data integrity and facilitate accurate assessment and documentation. The development of the MDS therefore commenced with an examination of the existing wound documentation standards of the participating healthcare organizations. Both standards are similar in structure and content, with only minor differences in terminology and specific domains. These differences were addressed by the expert panel, which represented both university hospitals. A notable variation exists in the PU/PI classification systems implemented at each site. While one hospital utilizes the ICD-10-GM system, the other employs the EPUAP framework. Despite their differences, both systems share considerable similarities. It is important to note that within the German hospital setting, the ICD-10-GM is the predominant and officially recognized classification system [3, 40]. Consequently, we included the ICD-10-GM in the MDS due to its relevance to the German healthcare context.

Our findings highlight a significant gap in clinical practice with regard to the documentation and assessment of IAD. Neither of the participating university hospitals had implemented a standardized assessment instrument or a structured data format for documenting IAD. This lack of standardization underscores the challenges of consistent and reliable data collection.

Our reliability analysis of wound image assessments revealed that inter- and intrarater agreement for IAD categorization was consistently lower than for PU/PI categorization. This highlights the need for standardized tools and training to improve the consistency of IAD assessments. The systematic assessment of IAD and its risk using a valid and reliable classification tool is strongly recommended by researchers and experts [4]. The identified best practice recommendation for IAD management is based on the GLOBIAD Categorization Tool [13]. The GLOBIAD Tool demonstrated robust content and face validity through the consensus of a large international panel of experts, with unanimous agreement on the description of the clinical presentation of IAD [9]. The findings of a recent systematic review of guidelines and consensus statements on IAD [41] corroborate our results. The review identified 10 relevant records, with five published prior to 2019 and two that are currently unavailable for analysis. Among the remaining three, one is a medical guideline on incontinence that offered limited insights into IAD management. The other two records, including the best practice recommendation from the Wund-D.A.CH. umbrella organization identified in our study, both endorsed the GLOBIAD Categorization Tool for documentation and categorization [13, 42]. The integration of a validated assessment tool not only enhances the accuracy of IAD assessment and documentation but also minimizes the misclassification of PU/PI, fungal and erythema skin conditions [43].

The wound images for AI training were derived from routine care. As we expected a considerable proportion of incorrect and incomplete routine documentations, we established a comprehensive validation process. Wound characteristics were independently annotated by a group of four experts following the MDS. We conducted a consensus discussion among the experts to resolve discrepancies in their assessments. Only images for which at least two wound experts agreed on their annotations were included in the data set. This rigorous process ensured the accuracy and consistency of the annotations, providing a reliable foundation for training and testing the AI system. The differentiation between PU/PI and IAD depends primarily on aetiology, anatomical location, and wound morphology [3, 36]. These differentiating factors extend beyond mere photographic features, encompassing both visual and contextual patient data. This led to the comprehensive integration of wound image data combined with categorical patient information for AI training, which has not been reported in previous studies, thereby enhancing the classification performance of the system [31].

The interrater reliability analysis demonstrated substantial to almost perfect agreement for wound type classification, indicating a high level of consistency among raters. However, this consistency was notably lower in batch 4, which had the fewest cases, suggesting that sample size may influence agreement levels. Excluding the category ‘both wound types’ from the analysis resulted in improved reliability. This finding can likely be attributed to the lack of a standardized instrument for this category and its limited use in clinical practice, making it more challenging for raters to achieve consensus. The analysis also revealed that agreement for IAD categorization was the weakest, particularly in batches 1 and 4, where agreement was only poor to slight. This finding likely stems from the limited training and experience of the annotators in using the GLOBIAD tool, as the reliability of assessments is closely tied to the level of education and expertise of the evaluators [9]. Enhancing the training of annotators could significantly strengthen the reliability of IAD assessments, thereby improving the overall quality of the data set used for AI training. Additionally, the difficulty in assessing clinical signs of infection based solely on photographic evidence is especially challenging [9], further contributing to the observed variability in agreement. The performance of AI depends heavily on large, carefully labeled data sets. However, data labeling can be a complex task even for experienced domain experts. These inconsistencies, characterized by high inter- and intra-observer variability, are referred to as ‘noisy labels’ [44, 45]. Despite the noisy labels that may resulted from this variability, a deep learning model trained on such data can perform as effectively as human clinicians [46]. This can be attributed to the ability of large deep neural networks to learn dominant patterns in the data set during training, rather than merely memorizing the data, allowing them to perform robustly despite some label noise. Chang et al. demonstrated the ability of a deep learning (DL) algorithm to monitor PU/PI healing [47]. Cicceri et al. showed encouraging results with a posture recognition tool based on DL techniques in the prevention of PU/PI [48]. Veredas et al. proposed a neural network system to detect PU/PI and classify wound tissue with considerable accuracy [49]. Available research on irritant contact dermatitis and AI is scarce. Mc Mullen et al. identified six studies in the existing literature up to early 2024 on machine learning (ML) applied to contact dermatitis [50]. Three of these studies used image data to train ML models to differentiate between allergic and irritant contact dermatitis. These studies reported diagnosis accuracies above 90%.

While our study provides valuable insights into the development of the AI-based decision support system, several limitations should be noted. First, the literature search was limited to the last five years, and it is possible that additional relevant studies may have been identified in a more comprehensive search. Second, the PU/PI categorization of suspected deep tissue injury as at least stage 3 according to ICD 10, while a separate classification is made according to EPUAP, may have led to a disproportionate weighting of stage 3. Finally, the data set may not fully capture the diversity of clinical environments and patient populations, which could restrict the applicability of the findings to different contexts or demographic groups.

## Conclusions

The systematic integration of evidence from nursing science and practice in the development of the AI system contributed to a novel approach in the field, underscoring the critical role of evidence-based nursing knowledge in shaping innovative solutions for wound care. The adoption of a mixed methods approach facilitated the continuous integration of interdisciplinary perspectives, literature evidence and expert knowledge, thereby benefiting the development process.

In the domain of wound management, the integration of a standardized framework for wound categorization and documentation into the MDS is crucial for improving the reliability and effectiveness of the AI-based decision support system. By incorporating validated classification systems like GLOBIAD, the MDS can ensure that the AI is trained on high-quality, consistent data, which is essential for generating accurate and reliable predictions. Furthermore, the adoption of standardized tools in clinical practice can help bridge the gap between current practices and best practice recommendations, ultimately improving patient outcomes. AI-based decision support systems can facilitate this adoption by providing healthcare professionals with real-time, evidence-based guidance, thereby enhancing the practical implementation in daily clinical routines.

The annotated data set will serve as a critical resource for the development and refinement of the AI model, enabling it to accurately predict wound characteristics and differentiate between wound types. The accuracy of the AI model is currently being pilot tested in real-world settings at the Point-of-Care in both university hospitals, providing valuable insights into its practical application and effectiveness in clinical environments, with a sub-study investigating the effects on nursing-related outcomes [51].

## Data Availability

Due to privacy concerns, the data set used in this study cannot be shared publicly.

## Abbreviations

AI: Artificial Intelligence
DL: Deep Learning
EPUAP: European Pressure Ulcer Advisory Panel
GLOBIAD: Ghent Global IAD Categorization Tool
IAD: Incontinence-associated Dermatitis
ICD-10-GM: International Classification of Diseases, 10th Revision – German Modification
MDS: Minimum Data Set
ML: Machine Learning
PU/PI: Pressure Ulcer/Pressure Injury
TRIPOD-AI: Transparent Reporting of a multivariable prediction model for Individual Prognosis or Diagnosis – Artificial Intelligence
Wund-D.A.CH: Umbrella Organization of German-speaking Wound Treatment Societies (Germany, Austria, Switzerland)

## Footnotes

1 Title combines German acronyms for artificial intelligence (KI), incontinence-associated dermatitis (IAD) and pressure ulcer/pressure injury (DEKU).

## References

[1] Beeckman D, Schoonhoven L, Fletcher J, Furtado K, Gunningberg L, Heyman H, et al. EPUAP classification system for pressure ulcers: European reliability study. J Adv Nurs. 2007;60(6):682–91. 18039255 10.1111/j.1365-2648.2007.04474.x

[2] Beeckman D, Schoonhoven L, Fletcher J, Furtado K, Heyman H, Paquay L, et al. Pressure ulcers and incontinence-associated dermatitis: Effectiveness of the pressure ulcer classification education tool on classification by nurses. Qual Saf Health Care. 2010;19(5):–. 20671078 10.1136/qshc.2008.028415

[3] Kottner J, Kolbig N, Bültemann A, Dissemond J. Incontinence-associated dermatitis: A position paper. Hautarzt. 2020;71:46–52. 31538217 10.1007/s00105-019-04480-7

[4] Banharak S, Panpanit L, Subindee S, Narongsanoi P, Sanun-Aur P, Kulwong W, et al. Prevention and care for incontinence-associated dermatitis among older adults: A systematic review. J Multidiscip Healthc. 2021:2983–3004. 10.2147/JMDH.S329672 PMC8556723 34729012

[5] Haesler E. European pressure ulcer advisory panel, national pressure injury advisory panel and pan pacific pressure injury alliance. EPUAP/NPIAP/PPPIA. 2019.

[6] Edsberg LE, Black JM, Goldberg M, McNichol L, Moore L, Sieggreen M. Revised national pressure ulcer advisory panel pressure injury staging system: Revised pressure injury staging system. J Wound Ostomy Continence Nurs. 2016;43(6):585–97. 27749790 10.1097/WON.0000000000000281 PMC5098472

[7] Büscher A, Blumenberg P, Krebs M, Schiemann D, Stehling H. Expertenstandard dekubitusprophylaxe in der pflege. 2. Aktualisierung (Juni 2017). ed. Osnabrück: Deutsches Netzwerk für Qualitätsentwicklung in der Pflege (DNQP); 2017. p.S1–116.

[8] Gefen A, Brienza DM, Cuddigan J, Haesler E, Kottner J. Our contemporary understanding of the aetiology of pressure ulcers/pressure injuries. Int Wound J. 2022;19(3):692–704. 34382331 10.1111/iwj.13667 PMC8874092

[9] Beeckman D, Van den Bussche K, Alves P, Arnold Long MC, Beele H, Ciprandi G, et al. Towards an international language for incontinence-associated dermatitis (IAD): Design and evaluation of psychometric properties of the ghent global IAD categorization tool (GLOBIAD) in 30 countries. Br J Dermatol. 2018;178(6):1331–40. 29315488 10.1111/bjd.16327

[10] Defloor T, Schoonhoven L, Katrien V, Weststrate J, Myny D. Reliability of the European pressure ulcer advisory panel classification system. J Adv Nurs. 2006;54(2):189–98. 16553705 10.1111/j.1365-2648.2006.03801.x

[11] Weir D. Pressure ulcers: Assessment, classification, and management. Chronic wound care: A clinical source book for healthcare professionals. In: Communications.4th. Malvern, PA: HMP; 2007. p. 575–81.

[12] Al Mutairi KB, Hendrie D. Global incidence and prevalence of pressure injuries in public hospitals: A systematic review. Wound Med. 2018;22:23–31.

[13] Dissemond J, Assenheimer B, Gerber V, Hintner M, Puntigam MJ, Kolbig N, et al. Moisture-associated skin damage (MASD): A best practice recommendation from Wund-D.A.CH. J Dtsch Dermatol Ges. 2021;19(6):815–25. 33942514 10.1111/ddg.14388

[14] Cunich M, Barakat-Johnson M, Lai M, Arora S, Church J, Basjarahil S, et al. The costs, health outcomes and cost-effectiveness of interventions for the prevention and treatment of incontinence-associated dermatitis: A systematic review. Int J Nurs Stud. 2022;129:104216. 35364428 10.1016/j.ijnurstu.2022.104216

[15] Patton D, Moore ZE, Boland F, Chaboyer WP, Latimer SL, Walker RM, et al. Dressings and topical agents for preventing pressure ulcers. Cochrane Database Syst Rev. 2024:1469–493X (Electronic). 10.1002/14651858.CD009362.pub4 PMC11613325 39625073

[16] Tomova-Simitchieva T, Akdeniz M, Blume- Peytavi U, Lahmann N, Kottner J. Die epidemiologie des dekubitus in Deutschland: Eine systematische übersicht. Gesundheitswesen. 2019;81(06):505–12. 29329470 10.1055/s-0043-122069

[17] Lichterfeld-Kottner A, Hahnel E, Blume-Peytavi U, Kottner J. Systematic mapping review about costs and economic evaluations of skin conditions and diseases in the aged. J Tissue Viability. 2017;26(1):6–19. 27544020 10.1016/j.jtv.2016.07.002

[18] Raepsaet C, Fourie A, Van Hecke A, Verhaeghe S, Beeckman D. Management of incontinence-associated dermatitis: A systematic review of monetary data. Int Wound J. 2021;18(1):79–94. 33236846 10.1111/iwj.13496 PMC7948709

[19] Demarré L, Van Lancker A, Van Hecke A, Verhaeghe S, Grypdonck M, Lemey J, et al. The cost of prevention and treatment of pressure ulcers: A systematic review. Int J Nurs Stud. 2015;52(11):1754–74. 26231383 10.1016/j.ijnurstu.2015.06.006

[20] Naylor CD. On the prospects for a (deep) learning health care system. Jama. 2018;320(11):1099–100. 30178068 10.1001/jama.2018.11103

[21] Anisuzzaman D, Wang C, Rostami B, Gopalakrishnan S, Niezgoda J, Yu Z. Image-based artificial intelligence in wound assessment: A systematic review. Adv Wound Care. 2022;11(12):687–709. 10.1089/wound.2021.0091 34544270

[22] Ganesan O, Morris MX, Guo L, Orgill D. A review of artificial intelligence in wound care. AIS. 2024;4(4):364–75.

[23] Jiang M, Ma Y, Guo S, Jin L, Lv L, Han L, et al. Using machine learning technologies in pressure injury management: Systematic review. JMIR Med Inform. 2021;9(3):e25704. 33688846 10.2196/25704 PMC7991995

[24] Seibert K, Domhoff D, Bruch D, Schulte-Althoff M, Fürstenau D, Biessmann F, et al. Application scenarios for artificial intelligence in nursing care: Rapid review. J Med Internet Res. 2021;23(11):e26522. 34847057 10.2196/26522 PMC8669587

[25] BfArM. German clinical trials register: The federal institute for drugs and medical devices (bundesinstitut für arzneimittel und medizinprodukte, BfArM); 2023 [Available from: https://drks.de/search/en/trial/DRKS00029961.

[26] Nordhausen T, Hirt J. Manual zur literaturrecherche in fachdatenbanken. Ref Hunter, Version. 2020:3.

[27] World Health Organization. ICD-10 (version: 2019) 2019 [updated 25.12.2024. Available from: https://icd.who.int/browse10/2019/en#/L89.

[28] Hayes AF, Krippendorff K. Answering the call for a standard reliability measure for coding data. Commun Methods Meas. 2007;1(1):77–89.

[29] Landis JR, Koch GG. The measurement of observer agreement for categorical data. Biometrics. 1977;33(1):159–74. 843571

[30] Artstein R, Poesio M. Inter-coder agreement for computational linguistics. Comput Linguist. 2008;34(4):555–96.

[31] Brehmer A, Seibold C, Egger J, Majjouti K, Tapp-Herrenbrueck M, Pinnekamp H, et al. Fine-grained classification of pressure ulcers and incontinence-associated dermatitis using multimodal deep learning: Algorithm development and validation study. JMIR AIforthcoming.2025. 10.2196/67356 PMC12223690 40605794

[32] Collins GS, Moons KGM, Dhiman P, Riley RD, Beam AL, Van Calster B, et al. TRIPOD+AI statement: Updated guidance for reporting clinical prediction models that use regression or machine learning methods. Bmj. 2024;385:e078378. 38626948 10.1136/bmj-2023-078378 PMC11019967

[33] Registered Nurses’ Association of Ontario. Assessment and management of pressure injuries for the interprofessional team. Toronto: Registered Nurses’: Association of Ontario;2016.

[34] Healthcare Improvement Scotland. Prevention and management of pressure ulcers standards October 2020 2020.

[35] National Clinical Guideline C. National institute for health and care excellence: Guidelines. The prevention and management of pressure ulcers in primary and secondary care. London: National Institute for Health and Care Excellence (NICE);2014. 25340232

[36] Beeckman D, Campbell J, Campbell K, Chimentao D, Coyer F, Domansky R, et al. Incontinence-associated dermatitis: Moving prevention forward: Addressing evidence gaps for best practice (proceedings from the global IAD expert panel). Wounds International 2015. 2015.

[37] Coleman S, Nelson EA, Vowden P, Vowden K, Adderley U, Sunderland L, et al. Development of a generic wound care assessment minimum data set. J Tissue Viability. 2017;26(4):226–40. 29030056 10.1016/j.jtv.2017.09.007

[38] Deutsche Gesellschaft für Wundheilung und Wundbehandlung e. V. S3-guideline 091–001: Local therapy of non-healing and/or chronic wounds due to peripheral arterial disease, diabetes mellitus, or chronic venous insufficiency. Version. 2023;2(2):31stOctober.

[39] DGfW. Lokaltherapie schwerheilender und/oder chronischer wunden aufgrund von peripherer arterieller verschlusskrankheit, diabetes mellitus oder chronischer venöser insuffizienz. Deutsche Gesellschaft Für Wundheilung Und Wundbehandlung e.V. DGfW); 2023.

[40] Federal Institute for Drugs and Medical Devices. ICD-10-GM - international statistical classification of diseases, German Modification: Federal Institute for Drugs and Medical Devices (BfArM); 2025 [Available from: https://www.bfarm.de/EN/Code-systems/Classifications/ICD/ICD-10-GM/_node.html.

[41] Chen Y, Gao Y, Zhang J, Niu M, Liu X, Zhang Y, et al. Quality and clinical applicability of recommendations for incontinence-associated dermatitis: A systematic review of guidelines and consensus statements. J Clin Nurs. 2023;32(11-12):2371–82. 35411654 10.1111/jocn.16306

[42] Fletcher J, Beeckman D, Boyles A, Fumarola S, Kottner J, McNichol L, et al. International best practice recommendations: Prevention and management of moisture-associated skin damage (MASD). 2020.

[43] Montague M, Karafa M, Albert NM. Assessment and documentation of incontinence-associated dermatitis after implementation of a standardised instrument: A comparative study. J Wound Care. 2019;28(Sup9):S4-S11. 31509491 10.12968/jowc.2019.28.Sup9.S4

[44] Song H, Kim M, Park D, Shin Y, Lee JG. Learning from noisy labels with deep neural networks: A survey. IEEE Trans Neural Netw Learn Syst. 2023;34(11):8135–53. 35254993 10.1109/TNNLS.2022.3152527

[45] Karimi D, Dou H, Warfield SK, Gholipour A. Deep learning with noisy labels: Exploring techniques and remedies in medical image analysis. Med Image Anal. 2020;65:101759. 32623277 10.1016/j.media.2020.101759 PMC7484266

[46] Ramachandram D, Ramirez-garcialuna JL, Fraser RD, Martínez-Jiménez MA, Arriaga-Caballero JE, Allport J. Fully automated wound tissue segmentation using deep learning on mobile devices: Cohort study. JMIR Mhealth Uhealth. 2022;10(4):e36977. 35451982 10.2196/36977 PMC9077502

[47] Chang CW, Christian M, Chang DH, Lai F, Liu TJ, Chen YS, et al. Deep learning approach based on superpixel segmentation assisted labeling for automatic pressure ulcer diagnosis. PLoS One. 2022;17(2):e0264139. 35176101 10.1371/journal.pone.0264139 PMC8853507

[48] Cicceri G, De Vita F, Bruneo D, Merlino G, Puliafito A. A deep learning approach for pressure ulcer prevention using wearable computing. HCIS. 2020;10(1):5.

[49] Veredas FJ, Luque-Baena RM, Martín-Santos FJ, Morilla-Herrera JC, Morente L. Wound image evaluation with machine learning. Neurocomputing. 2015;164:112–22.

[50] McMullen E, Grewal R, Storm K, Maazi M, Butt AB, Gupta R, et al. Diagnosing contact dermatitis using machine learning: A review. Contact Dermatitis. 2024. 10.1111/cod.14595 38831517

[51] Pinnekamp H, Rentschler V, Majjouti K, Brehmer A, Tapp-Herrenbrück M, Aleithe M, et al. Controlled pilot intervention study on the effects of an AI-based application to support incontinence-associated dermatitis and pressure injury assessment. Nurs Care Doc: Study Protocol. Res Nurs Health. 2025;n/a(n/a). 10.1002/nur.22469 PMC12217424 40237306

